# Coordinated balance of Rac1 and RhoA plays key roles in determining phagocytic appetite

**DOI:** 10.1371/journal.pone.0174603

**Published:** 2017-04-04

**Authors:** Sang-Yeob Kim, Soyoun Kim, Dong-Jun Bae, Seung-Yoon Park, Ga-Young Lee, Gyeong-Min Park, In-San Kim

**Affiliations:** 1 Department of Biochemistry and Cell Biology, Cell and Matrix Research Institute, School of Medicine, Kyungpook National University, Daegu, Republic of Korea; 2 ASAN Institute for Life Sciences, ASAN Medical Center, Seoul, Republic of Korea; 3 Department of Convergence Medicine, University of Ulsan College of Medicine, Seoul, Republic of Korea; 4 Department of Biochemistry, School of Medicine, Dongguk University, Gyeongju, Republic of Korea; 5 Biomedical Research Institute, Korea Institute of Science and Technology, Seoul, Republic of Korea; 6 KU-KIST school, Korea University, Seoul, Republic of Korea; Leiden University, NETHERLANDS

## Abstract

The removal of unwanted or damaged cells by phagocytes is achieved via a finely regulated cleaning process called efferocytosis. To characterize the mechanisms through which phagocytes control the intake of apoptotic cells, we investigated how the phagocyte’s appetite for engulfed cells may be coordinated by RhoA and Rac1 in the phagocytic cup. We used FRET biosensors to visualize the spatiotemporal dynamics of Rho-family GTPases, and found that RhoA, which is known to be downregulated during phagocytosis, was transiently upregulated at the phagocytic cup immediately prior to ingestion. Conversely, Rac1 was upregulated during the engulfment process and then downregulated prior to phagosomal maturation. Moreover, disturbance of the dynamic activities of RhoA led to uncontrolled engulfment, such as fast and undiscerning eating. Our results reveal that the temporal activity of RhoA GTPase alters the Rac1/RhoA balance at the phagocytic cup prior to ingestion, and that this plays a distinct role in orchestrating efferocytosis, with RhoA modulating the rate of engulfment to ensure that the phagocyte engulfs an appropriate amount of the correct material.

## Introduction

Efferocytosis, which is the process through which the body clears unwanted or damaged cells, is essential for development, homeostasis, and injury responses. This cellular process must be efficient and precise, since failed or defective clearance has been associated with a range of disease processes, including atherosclerosis, chronic inflammation, and autoimmunity [1]. Dying cells are recognized and promptly removed by neighboring or professional phagocytes via sequential phagocytic processes that include recognition, engulfment, and degradation [2–5]. When phagocytes are near to apoptotic cells, specific interaction is mediated between engulfment receptors on phagocytes and phosphatidylserine (PS), one of “eat-me” signals, on the apoptotic cell surface. The internalization of the apoptotic cells are processed by F-actin rearrangement forming a phagocytic cup, a cup-shaped invaginations of the cell membrane that subsequently close at their distal margins to form phagosomes during phagocytosis. The phagosome is processed to its degradation. This maturation step involves the release of anti-inflammatory cytokines to mediate the immunologically silent removal of apoptotic cells [6]. Each step must be finely regulated to avoid non-selective or uncontrolled “eating” (phagocytosis) of cells, but we do not yet understand the precise molecular mechanisms that enable phagocytes to control their eating and digesting. This so-called “appetite control” needs to be able to: 1) discriminate between viable and dead/dying cells; and 2) control engulfment with respect to the capacity of the phagocyte to prevent overeating. A phagocyte’s ultimate decision to ingest a cell is reflected by closure of the phagocytic cup (engulfment), which occurs via dynamic rearrangement of the cytoskeleton, and is controlled by the Rho-family GTPases [7–11]. This mechanical movement of membrane participates specific irreversible steps in the phagocytic process thus must be controlled to ensure that a proper target is engulfed at the correct rate and amount.

Genetic and biochemical studies in *C*. *elegans* and mammals have revealed that the Rho-family GTPases, RhoA and Rac1, are antagonistically involved in modulating cytoskeletal reorganization during efferocytosis. Their specific actions have not yet been fully elucidated, but studies using dominant-negative (DN) or constitutively activated (CA) mutants of RhoA and Rac1 [12, 13] have indicated that Rac1 and its upstream activators facilitate the engulfment of apoptotic cells, whereas RhoA and its downstream effector, Rho kinase (ROCK), have inhibitory functions. The phagocytic signals which are triggered by the PS-receptor interactions converge at Rac1 activation, in turn, to activate actin polymerization on the specific membrane sites. Since the Rho-family GTPases are known to act as molecular “switches” that can turn signaling pathways on and off by cycling from the GTP-bound active state to the GDP-bound inactive state, Rac1 can modulate actin rearrangement by turning on/off its activity. The activity of Rac1 in the phagocytic membrane is decreased, accompanied by F-actin disassembly and the closure of the phagocytic cup [14, 15]. Evidences of inhibitory function of RhoA via ROCK in phagocytosis are accumulated but how and when the RhoA/ROCK pathway is activated and how it inhibits the engulfment of apoptotic cells is not fully elucidated [12, 16, 17](12, 16, 17)(12, 16, 17)(12, 16, 17). Erwig *et al*. reported that the RhoA/ROCK phosphorylates ezrin-radixin-moesin (ERM) proteins and play roles in regulation of phagosome maturation in macrophages and dendritic cells [18].

Here, we used FRET-based biosensors to perform real-time comparisons of Rac1 and RhoA in phagocytes during the uptake of apoptotic cells (i.e., during formation and closure of the phagocytic cup). We unexpectedly found that RhoA was transiently upregulated at the phagocytic cup immediately prior to ingestion and hypothesized that this active RhoA may modulate engulfment prior to closure of the phagocytic cup, thus allowing time for engulfment decisions to prevent non-selective engulfment or overeating, beyond the traditional concept of simple antagonistic regulation. Our results suggest that dynamic Rac1/RhoA balance in the phagocytic cup modulate the critical moment at which a phagocyte decides whether to proceed with eating a meal. We believe that our findings provide novel insights into one of fundamental questions regarding phagocytosis; namely, how the appetite of a phagocyte is controlled through cytoskeleton modulation mediated by the dynamic balance of Rac1 and RhoA.

## Materials and methods

### Reagents

L-phosphatidylserine (PS, 840032), L-phosphatidylcholine (PC, 840055), 1-oleoyl-2-{6-[(7-nitro-2-1,3-benzox-adiazol-4-yl)amino]hexanoyl}-sn-glycero-3-phosphocholine (NBD-PC, 810132) were purchased from Avanti-Polar Lipids. Y27632 (688000) was purchased from Calbiochem. Anti-human CD16 (clone: 3G8, 555404), anti-human CD32 (clone: 3D3, 551900), and human CD47-Fc (clone: B6H12, 556044) were purchased from BD Biosciences.

### Constructs

Fluorescence resonance energy transfer (FRET)-based Rac1 (Raichu-Rac1/K-Ras–CT) and RhoA (Raichu-RhoA/K-Ras–CT) probes were kindly provided by Prof. M. Matsuda (Laboratory of Bioimaging and Cell Signaling, Graduate School of Biostudies, Kyoto University, Kyoto, Japan) [12, 19, 20]. The cDNAs of Rac1 and RhoA were amplified by PCR and inserted into the *BamHI*/*EcoRI* sites of pcDNA3. Rac1G12V, Rac1T17N, RhoAG14V, and RhoAT19N were generated by modified QuikChange mutagenesis [21]. The pCAG-myc-p160D3 was kindly provided by Prof. Shuh Narumiya (Department of Pharmacology, Kyoto University Graduate School of Medicine, Kyoto, Japan) [22]. The cDNAs of p160ROCK D3 was amplified and inserted into the *KpnI*/*XhoI* sites of pcDNA3.

### Cell cultures

L cells that were stably transfected with stabilin-2-Myc (L/Stab-2 cells) were maintained as previously reported [23, 24]. Human monocyte-derived macrophages (HMDMs) were obtained as described [23]. In brief, human monocytes were obtained by standard protocols from buffy coats from healthy donors. The experiment procedures using human blood samples were performed in compliance of the institutional guidelines and were approved by the Institutional Review Board (IRB) of Kyungpook National University (permission No. KNUBIO 07–1006). The participants have been properly instructed and signed the informed consent forms. The procedure was performed under the guidance of IRB of Kyungpook National University.

Cells were allowed to adhere and differentiate for 10 days at 37°C under 5% CO_2_. Mouse peritoneal macrophages were isolated from 6-to-8-week-old male Balb/c mice 4 days after the intraperitoneal injection of 3% Brewer thioglycollate medium (1 ml) and maintained in RPMI-1640 medium containing 10% (v/v) fetal bovine serum (FBS) and antibiotics[25]. Bone marrow-derived macrophages (BMDMs) were isolated from 6-to-8-week-old male Balb/c mice and treated with red blood cell lysis buffer [26]. The suspended cells were cultured with 20 ng/ml macrophage colony-stimulating factor (M-CSF) for 5 days. Mouse cells were collected with institutional guidelines and according to the animal protocol approved based on the guidelines of the Institutional Animal Care and Use Committee (IACUC) of Kyungpook National University (permission No. KNU 2012 48).

### Transfection

L/Stab-2 cells were plated at a density of 5×10^4^ cells into collagen-coated 24-wells plates and grown to 90% confluence. To study the effects of Rac1, RhoA and ROCK on phagocytosis, L/Stab-2 cells were transfected with empty vector, Rac1G12V, Rac1T17N, RhoAG14V, RhoAT19N, and p160ROCK **Δ**3 for 16–18 h using Lipofectamine 2000 (Invitrogen) according to the manufacturer’s instructions. Twenty-four hours later, the engulfment of the PS-exposed RBCs was assayed. For FRET image analysis, L/Stab-2 cells were cultured on collagen-coated 35-mm glass-bottomed dishes (Asahi techno glass, Tokyo, Japan) and transfected with FRET-based indicators using Lipofectamine 2000 (Invitrogen).

### Preparation of phospholipid-coated beads, PS-exposed damaged RBCs, and apoptotic/damaged cells

The fluorescence-labeled PS-coated beads were generated as previously described [27]. Briefly, Nucleosil 120–3 C18 beads (3 μm, 5 μm; Richard Scientific) were dissolved in chloroform, after which a mixture of PC:PS:NBD-PC (45:50:5 mol%) was added, and the suspension was dried under nitrogen gas. The beads were rehydrated with PBS and briefly sonicated before use. PS-exposed damaged RBCs were prepared by incubation in PBS (20% hematocrit) at 37°C for 4–5 days as previously described [23]. Thymocytes were isolated from the thymus of 6-to-8-week-old male Balb/c mice and treated for 4 h with 50 μM dexamethasone to induce apoptosis as previously described [12, 14].

### Active small GTPase pulldown assays

L/Stab-2 cells treated with PS liposomes for the indicated times were subjected to Rac1-GTP or RhoA-GTP pulldown assays using GST-PBD (kit #6118, Pierce) or GST-RBD (kit #16116, Pierce), respectively, as described previously[12]. The levels of Rac1-GTP or RhoA-GTP in the pulldowns and the total Rac1 or RhoA in the whole lysates were compared by western blot.

### Time-lapse FRET imaging

FRET images were obtained as described before [28]. Nikon Ti-E inverted microscope equipped with PFS and a CoolSNAP HQ camera (Roper Scientific) with excitation and emission filter wheels was employed. Images were acquired by using the 4×4 binning mode and a 200-ms exposure time. For dual-emission ratio imaging of the intramolecular FRET probe, we obtained images for CFP and FRET. Pseudo-color images of FRET/CFP were created using eight colors from red to blue to represent the FRET/CFP ratio via the intensity modulated display (IMD) mode of MetaMorph software. The following filters used for the dual-emission ratio imaging were purchased from Semrock: an FF01-438/24-25 excitation filter, an FF458-Di02-25×36 dichroic mirror, and two emission filters, FF01-483/32-25 for CFP and FF01-542/27-25 for FRET.

### Live-cell imaging

L/Stab-2 cells (3×10^5^) were plated on a collagen-coated 35-mm glass-base dish (Asahi techno glass, Japan) and grown overnight at 37°C. After 24 h, the cell culture media was changed to phenol-red-free media containing 10% (v/v) FBS, and apoptotic thymocytes (1.5×10^7^) were added. For phase-contrast images, the cells were placed in an incubation chamber equipped with a time-lapse imaging system (BioStation IM; Nikon, Japan).

### In vitro phagocytosis assay

The engulfment of damaged RBCs was assayed as previously described[23]. Briefly, PS-exposed damaged RBCs were added to L/Stab-2 cells, and the treated cells were incubated at 37°C in 5% CO_2_ for 1 h. After the unbound RBCs were washed away, the uningested RBCs were lysed by the addition of deionized H_2_O for 10 sec, which was immediately replaced with DMEM medium as described previously[23]. The cells were fixed with methanol, stained with a Diff Quick staining kit (IMEB Inc.), and counted under brightfield microscopy. The engulfment of the PS-coated beads was assayed as described[27]. In brief, PS/PC-NBD beads (3 or 5 μm, 100 μl) were added to peritoneal macrophages, BMDMs, or HMDMs and then incubated for 1 h in the presence or absence of Y27632. The fluorescence of surface-bound beads was quenched with 1 ml of trypan blue (0.4%) for 5 min at room temperature followed by cold PBS. The phagocytic index was determined by the number of ingested RBCs or beads per cell under light and fluorescence microscopy. The percentage of phagocytes with the indicated number of ingesting cells was calculated. At least 500 cells were scored per well, and all experiments were repeated at least three times. To examine phagocytosis after the addition of CD47-Fc protein, HMDMs were pretreated with 30 μM ROCK inhibitor (Y27632) or DMSO for 30 min, and possible Fcγ receptor-mediated effects were blocked by the addition of anti-human CD16/32 (25 μg/ml) for 10 min before the addition of CD47. CD47-Fc (20 μg/ml) (BD Biosciences) or Fc-protein were immediately added and incubated for an additional 30 min at 37°C in 5% CO_2_, and the engulfment of PS/PC-NBD beads was assayed as described above. To avoid bias, all phagocytic indexes were counted by people who are blind in the assay group.

### In vivo phagocytosis assay of peritoneal macrophages

Mouse experiments were performed in accordance with national and institutional guidelines for animal care and the protocol was approved by the Institutional Animal Care and Use Committee (IACUC) of Kyungpook National University (Permit Number: KNU 2012–133). All surgery was performed under inhalational anesthesia (1%, w/v, isoflurane in 2 L oxygen) and all efforts were made to minimize suffering. Six-to-eight-week-old male Balb/c mice (n = 6) were intraperitoneally injected with 1 ml of sterilized 3% thioglycollate (Sigma-Aldrich). After 96 h, the randomly chosen mice (n = 3) were intraperitoneally injected with 300 μl of PBS containing 10 mg/kg Y27632 dissolved in DMSO (20 mg/ml). Control mice (n = 3) were injected with PBS. Two hours after injection, the mice were intraperitoneally injected with PS/PC-NBD beads (3 μm, 300 μl) and sacrificed after 1 h, and peritoneal exudate cells (PEC) were harvested by peritoneal lavage, using 5 ml of PBS as described[25]. The concentration of cells was counted using a hemocytometer under a microscope. PECs (3.0×10^6^) were plated in 6-well culture plates using a Cytospin protocol. The fluorescence of surface-bound beads was quenched with 1 ml of trypan blue (0.4%) for 5 min at room temperature followed by cold PBS. The phagocytic index was determined as the number of ingested beads per cell under brightfield and fluorescence microscopy. The percentage of phagocytes with the indicated number of ingesting cells was calculated. At least 500 cells were scored per well, and experiments were repeated at least three times.

## Results

### Real-time activities of Rho GTPases in phagocytes undergoing efferocytosis

**Fig 1:**
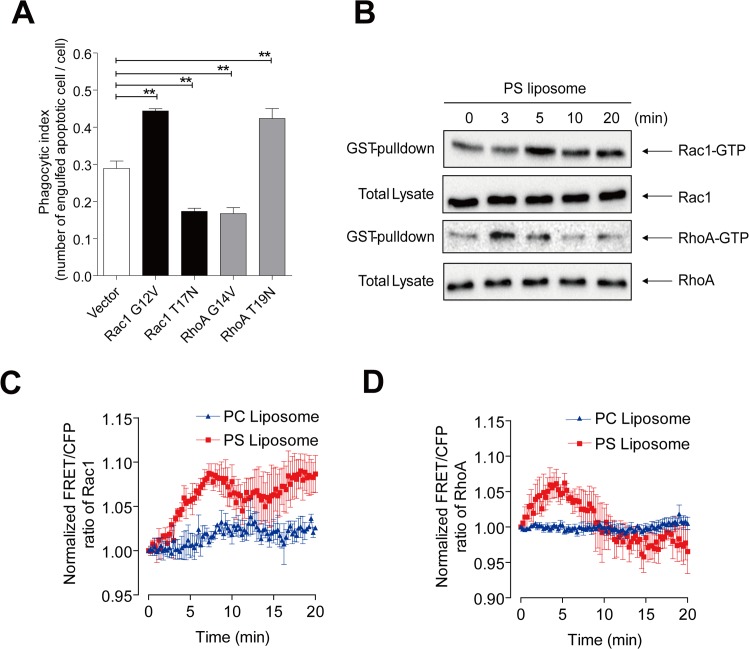
Activities of Rac1 and RhoA during phagocytosis by L/Stab-2 cells. (A) An L/Stab-2 cells were transfected with control vector, Rac1 G12V (CA), Rac1 T17N (DN), RhoA G14V (CA), or RhoA T19N (DN) mutants. After 1 h incubation with apoptotic thymocytes, the phagocytic index (number of engulfed apoptotic cell / Stab-2 cell) was calculated and shown as means + s.e.m. (n = 3 independent examinations per group, Each examination consists of >500 cells). (B) The Rac1-GTP and RhoA-GTP were pulled down from PS liposome-treated L/Stab-2 cells and analyzed by immunoblotting. (C,D) The FRET of Rac1 (C) and RhoA (D) after PC and PS liposome treatment (the error bars indicate the s.e.m. of the 3 experiments). The intensities were normalized to the value at 0 min.

Apoptotic cells are efficiently recognized by professional phagocytes, macrophages, and swiftly cleared without cause of inflammation. Many receptors function in the tethering of apoptotic cells via direct binding to the exposed PS or indirect binding through bridging molecules. Stabilin-2, one of PS binding receptors, is expressed in human monocyte derived macrophages (HMDMs) and mediates engulfment of apoptotic cells^19^. We employed L/Stab-2 cells, which stably express the PS receptor, stabilin-2, and thus efficiently engulf apoptotic cells [23, 29], to examine the specific dynamics of RhoA and Rac1 during phagocytosis of apoptotic cells. PS interacting stabilin-2 turns on the phagocytic signals which activate Rac1 in concert with adopter protein, GULP [24]. In agreement with previous observations in macrophages [13], overexpression of CA Rac1 (Rac1 G12V) promoted the phagocytosis of apoptotic thymocytes, whereas DN Rac1 (Rac1 T17N) inhibited this process (Fig 1A). Conversely, overexpression of CA RhoA (RhoA G14V) or DN RhoA (RhoA T19N) mutants yielded the opposite results, confirming that Rac1 and RhoA play positive and negative roles, respectively, in the phagocytosis of L/Stab-2 cells.

The GTP-bound active forms of endogenous Rac1 and RhoA were examined after cells were challenged with PS liposomes, which can function as a simplified target to trigger phagocytic signals while avoiding contamination by target-cell-derived Rho family proteins [23]. The pull-down assays showed that the level of endogenous Rac-GTP in phagocytes was increased after 5 min of incubation with PS liposomes and maintained for 20 min, but the level of Rho-GTP was increased at 3 min and began to decrease after 5 min (Fig 1B). The latter observation was unexpected, since Rho-GTP was previously reported to decrease after incubation with an apoptotic cell mimic beads [12]. However the Rho-GTP was assayed after 30–120 min incubation with a target in their experiment. To further examine the dynamic features of these GTPase activities, we expressed Rac1 or RhoA FRET biosensors in L/Stab-2 cells, which were then treated with PS liposomes. Upon activation of Rac1 or RhoA, the intramolecular binding of their GTP-bound forms to the Cdc42-Rac-interacting binding motif (CRIB) enhances FRET between YFP and CFP [12, 19]. Our real-time observations of the activities of Rac1 and RhoA were consistent with the results of our pull-down assay: Rac1 was activated after 5–20 min, while RhoA was transiently activated around 3 min (Fig 1C and 1D, S1 Fig). By challenge of PS liposomes, membrane motility is increased upon activation of Rac1, forming lamellipodia (S1 Fig). Comparing to Rac1, the PS-stimulated RhoA activity was mild and transient without correlation with membrane motility. The activities of Rac1 and RhoA were not influenced by challenge with phosphatidylcholine (PC) liposomes, suggesting that both activations require PS.

**Fig 2:**
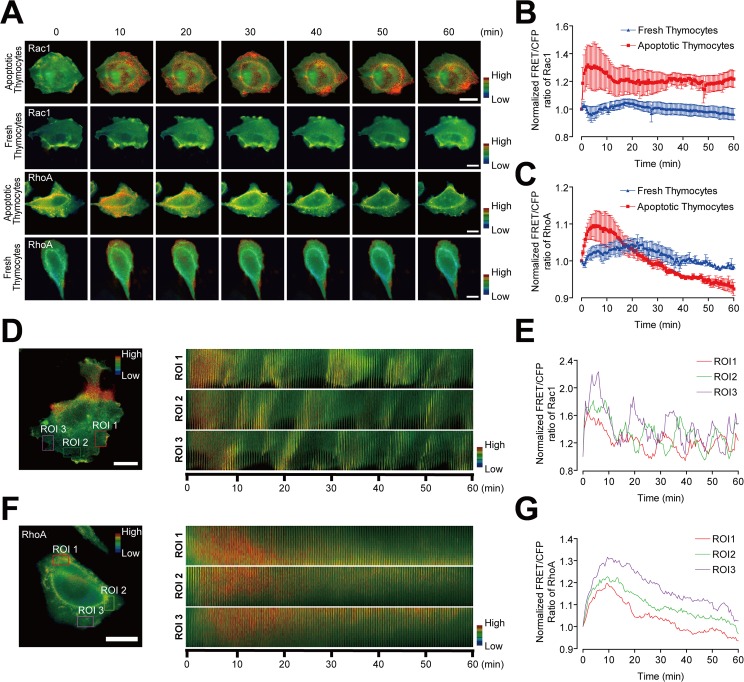
Real time activities of Rho GTPases in phagocytes upon challenge with apoptotic thymocytes. (A) Representative images of active Rac1 and RhoA biosensor-transfected L/Stab-2 cells treated with apoptotic or fresh thymocytes (Scale bar: 20 μm). (B,C) The normalized FRET of Rac1 (B) and RhoA (C) with apoptotic and fresh thymocytes (the error bar indicates the s.e.m. for the 3 experiments). (D-G) Three regions of interest (ROIs) from the Rac1 (D) and RhoA (F) biosensor-transfected cells are shown, along with the time (Scale bar: 20 μm). The FRET of Rac1 (E) and RhoA (F) in the indicated ROIs are shown. The intensities were normalized to the value at 0 min (B,C,E,G).

To examine real-time GTPase activities triggered by physiological targets, L/Stab-2 cells expressing Rac1 or RhoA biosensors were combined with excess number of mouse thymocytes in which apoptosis had been induced by dexamethasone treatment. The overall activity of Rac1 increased after the addition of apoptotic cells and remained elevated for the duration of the observation (60 min) (Fig 2A and 2B, S1 and S2 Movies). As like in PS liposome treatment, the Rac1 activity was mainly observed in lamellipodia, and close examination of three regions of interest (ROIs) revealed that Rac1 activity fluctuated vigorously upon membrane movement at multiple sites with synchronous waves, implying Rac1 activity is related with membrane motility (Fig 2D and 2E, S5 Movie). The overall activity level of RhoA was also elevated throughout the cells, not in the lamellipodia, peaking at 10 min and diminishing thereafter (Fig 2A and 2C, S3 and S4 Movies). This transient activation of RhoA was concurrently observed in several regions of L/Stab-2 cells following their exposure to apoptotic cells (Fig 2F and 2G, S6 Movie). Strong activation of Rac1 triggers actin polymerization, playing a positive role in the efficient uptake of apoptotic cells. However, this transient activation of RhoA was somewhat surprising, because RhoA acts as a negative regulator and was previously reported to be downregulated during phagocytosis [12, 13].

To attest the Rac1/RhoA activity is directly related with phagocytic process, separately from membrane motility, we monitored Rac1/RhoA FRET activities acquired after contact with an individual apoptotic cell, and chased the activities around the phagocytic cup of single ingested cell.

### The spatiotemporal activities of Rac1 and RhoA at the phagocytic cup during the engulfment of individual apoptotic cells

**Fig 3:**
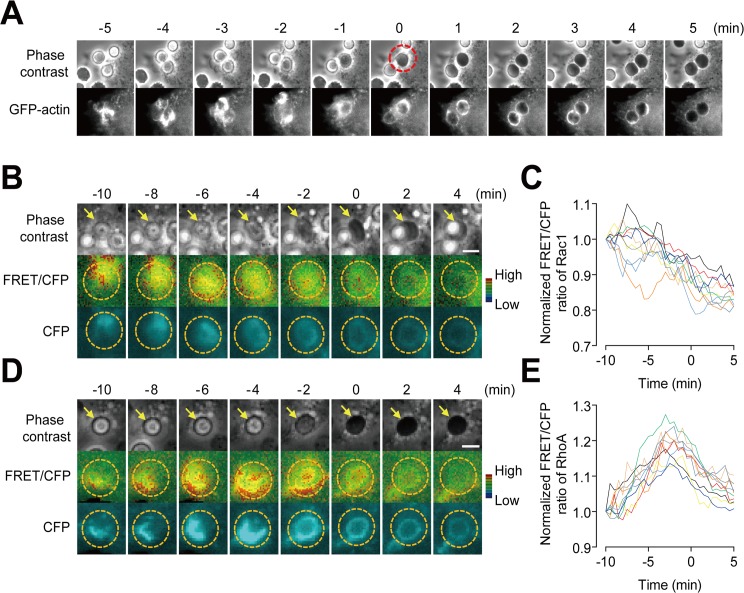
Activities of Rac1 and RhoA around apoptotic cells during engulfment. (A) Representative phase contrast (PC) and GFP-actin images after the addition of the PS-exposed RBCs into L/Stab-2 cells. Time 0 (closure of phagocytic cup) was defined as the point when the color of the apoptotic cell changed (red dotted circle). (B-E) The images represent the FRET of active Rac1 (B,C) and RhoA (D,E) around the RBC during engulfment (n = 10, Scale bar: 10 μm; yellow arrow points to the engulfed cell). The intensities of the Rac1 (C) and RhoA (E) biosensors were normalized to the value at -10 min.

To investigate the distinct spatiotemporal activities of Rac1 and RhoA during the engulfment of individual apoptotic cells, we monitored their FRET signals at the phagocytic cup during individual engulfment events (Fig 3). The regions of interest (yellow dotted circle) were selected enough to measure all FRET signals which are appeared in the phagocytic membrane surrounding apoptotic cells. The ingestion time was defined as “0 min” upon the closing of the phagocytic cup, when the color of the apoptotic cell in a phase contrast image changed from light to dark [12, 14] (Fig 3A). Consistent with previous findings [14], Rac1 activity was observed around the ingested apoptotic cell, supporting the hypothesis that Rac1 promotes the polymerization of actin to enable the membrane to protrude and surround a dead cell. The FRET efficiency of the Rac1 probe gradually decreased before the phagocytic cup was sealed (starting around -5 min), and actin disassembly was also observed at that time (Fig 3A–3C, S7 Movie). As in the measurement of whole cells (Fig 2A and 2C), RhoA was transiently activated at the phagocytic cup during every engulfment event (n = 10) (Fig 3D and 3E, S8 Movie), immediately prior to the closure of the phagosomal membrane (-6 to -2 min). Both of actual CFP and YFP intensities were simultaneously decreased after phagocytic cup is closed but the FRET efficiency (ratio of YFP and CFP) was able to indicate Rac1 or RhoA activities. We excluded the possibility of bleaching effect because no CFP change was observed in other area. The declined CFP signals in later acquisition (Fig 3B) may be explained by the fact that the local probe amount in the focal phagocytic ring is diminished after uptake since we used membrane attached and intramolecular Rac1/RhoA FRET probe (Raichu Rac1 or RhoA/K-rac-CT). To explain that RhoA, a negative regulator, was activated in the phagocytic cup upon stimulation with apoptotic cells, we hypothesize that active RhoA may contribute to delaying engulfment prior to closure of the phagocytic cup, thus allowing time for engulfment decisions to prevent non-selective engulfment or overeating.

### Disruption of RhoA activity causes accelerated engulfment

**Fig 4:**
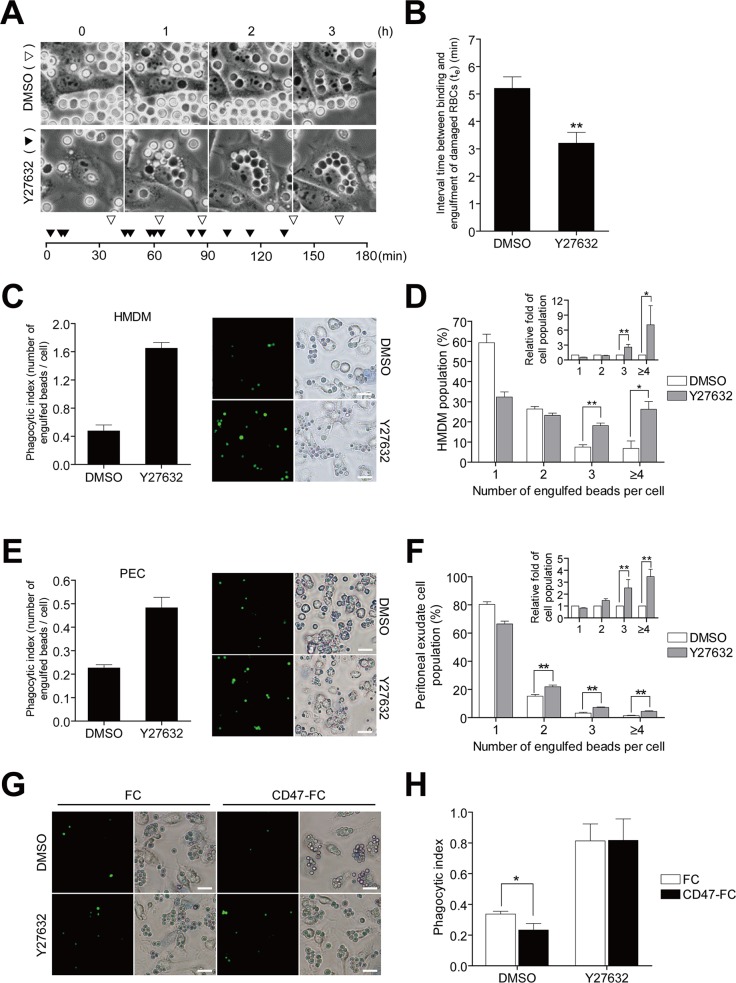
Accelerated and uncontrolled engulfment caused by inhibition of RhoA/Rho-kinase. (A) The engulfment of damaged RBCs by L/Stab-2 cells treated with the ROCK inhibitor (Y27632), as shown in DIC images (Scale bar, 20 μm). The ingestion time points of RBCs were plotted relative to time (bottom). ▽, DMSO-treated L/Stab-2 cells; ▼, Y27632-treated cells. (B) The time for engulfment (*t*_*e*_) was measured from 20 phagocytic events with and without Y27632. (C-F) The engulfment of NBD PS-coated beads by HMDMs (C) and peritoneal exudate cells (PECs) (E) is shown (Scale bar, 20 μm). The phagocytic index was determined as the average number of PS-coated beads ingested per cell. The percentages of HMDMs (D) and PECs (F) carrying the indicated numbers of beads were determined. The inset shows the relative populations compared to control. (G,H) CD47-Fc or Fc protein-preincubated HMDMs were tested for phagocytosis, shown as representative images (G) and a phagocytic index (H). The results are presented as the means + s.e.m. (n = 3 independent examinations per group; scale bar, 20 μm. Each examination consists of >500 cells.) *P<0.05, **P<0.01, unpaired Student’s *t*-test (B,C,E,H).

To test our hypothesis regarding this function of RhoA, we examined Rho kinase (ROCK); a primary effector of RhoA in phagocytosis during the engulfment of apoptotic cells by L/Stab-2 cells based on the results of RhoA effector mutants with L/Stab-2 cells. The RhoA CA mutant (G14V) is constitutively actable via various effector molecules and inhibits phagocytosis, but its negative function is lost by effector-defective RhoA mutant (G14V/T37Y) that cannot bind all effector molecules (S2 Fig). We observed that the ROCK/mDia-acting RhoA mutant (G14V/F39V), which can interact with mDia and ROCK but not the other known effectors was still able to inhibit engulfment of apoptotic cells. However, the G14V/F39A mutant that retains the ability to activate mDia, but not ROCK, completely lost its inhibitory effect on engulfment. This pointed to ROCK as a potential mediator of the inhibitory effect due to RhoA, as previously reported with J774 macrophages [12]. The phagocytic ability of these cells was also inhibited by overexpression of the CA forms of ROCK (p160ROCK D3). Moreover, the inhibitory effect of CA RhoA was reversed by the ROCK inhibitor, Y27632 (S2 Fig). Together, these findings suggest that ROCK is a primary effector of the function of RhoA in phagocytosis. To gain insight into ROCK-mediated RhoA function in phagocytosis, we monitored live Y27632-treated L/Stab-2 cells during the engulfment of PS-exposed red blood cells (RBCs) (Fig 4A). Vigorous engulfment was observed when ROCK was blocked, as shown in a plot of engulfment events over the time (Fig 4A bottom, S9 and S10 Movies), and the engulfment time (*t*_*e*_; the interval from attachment to ingestion at the same site) was substantially reduced (by approximately 40%) (Fig 4B). To determine whether this accelerated engulfment also occurs in primary macrophages, we examined the effect of ROCK inhibition on phagocytosis by human monocyte-derived macrophages (HMDMs). Indeed, treatment of HMDMs with Y27632 dramatically increased their phagocytic ability due to “overeating” as a consequence of rapid engulfment. Up to 42% of the Y27632-treated macrophages engulfed more than three beads, whereas only 16% of control macrophages showed that level of engulfment (Fig 4C and 4D). This tendency to overeat was also observed in L/Stab-2 cells and other macrophages, including mouse bone marrow-derived macrophages (BMDMs) and mouse peritoneal macrophages (S3 Fig). To assess the effect of ROCK inhibition *in vivo*, peritoneal exudate cells (PECs) were collected from mice injected with Y27632 and PS beads, and the engulfment of fluorescent-labeled PS beads was examined (Fig 4E and 4F). Similar to our *in vitro* observations, the PECs from Y27632-treated mice showed enhanced phagocytosis, reflecting a tendency toward overeating. This vigorous engulfment would be owing to Rac1 activation by Y27632. Rac1 and RhoA have been shown to exhibit mutual antagonism [30], mediated by p190Rho–GAP [31] or FilGAP [32]. We monitored Rac1/RhoA activation kinetics after treatment of Y27632 to L/Stab-2 cells (S4 Fig). Upon treatment of Y27632, Rac1 activity was elevated whereas RhoA activity was not affected.

Next, we investigated whether this increased eating was associated with uncontrolled phagocytosis (e.g., a lack of selectivity between living and dead cells). Specific cellular recognition events mediate signals that inhibit phagocytosis of viable cells. CD47, which is a “don’t eat me” signal found, on the surface of normal cells, is recognized by SIRPα on the phagocytes to initiate negative signals and block phagocytosis [33]. The addition of extracellular CD47 can also trigger this “don’t eat me” signal and inhibit host cell phagocytosis [34]. We found that treatment with CD47-Fc fusion protein decreased the phagocytic activity of control HMDMs, but not that of Y27632-treated HMDMs (Fig 4G and 4H), suggesting blockade of RhoA-ROCK signaling causes a loss of inhibition of phagocytosis in the presence of CD47. To clarify if CD47-Fc directly affects Rac1/RhoA activity, we monitored Rac1 and RhoA activation kinetics after treatment of CD47-Fc to L/Stab-2 cells which were transfected by Rac1/RhoA FRET probe (S5 Fig). The activity of Rac1 or RhoA of phagocytes was not affected by incubation with CD47-Fc, implying that CD47-SIRPα interaction does not activate RhoA nor suppress Rac1 to inhibit phagocytosis. We next examined if Y27632 also leads to uncontrolled engulfment of normal cells regardless of absent of eat-me signal, PS. We monitored Rac1 and RhoA activation kinetics after addition of PS or PC liposomes to the Y27632-pretreated phagocytes (S6 Fig). In Y27632-pretreated cells, PS liposomes triggers Rac1/RhoA activation in the similar pattern comparing in the Y27632 untreated condition, except that Rac1 activity appeared to be augmented. However, PC did not trigger any effects on Rac1/RhoA under Y27632 treated or untreated conditions, indicating inhibition of RhoA-ROCK did not lead to a loss of discrimination between PC and PS liposomes. This suggests that vigorous engulfment by Y27632 still relies on specific interaction of PS-receptor and eat-me signals. Overall, the blockade of RhoA function causes macrophages to engulf apoptotic cells too quickly, that the cells may ignore the anti-phagocytic signals. These data support our hypothesis that RhoA temporally inhibits engulfment of bound cells prior to ingestion, thus helping to ensure that phagocytes engulf only the correct meal.

## Discussions

Not just in efferocytosis, the Rho-family GTPases play key roles in controlling cytoskeletal dynamics and modulating the cell shape, division, migration, polarity, and adhesion [35]. The recent development of FRET biosensors that can be used to visualize the spatiotemporal dynamics of Rho-family GTPases has given researchers a more concrete sense of their roles in coordinating the cytoskeleton. For example, where Rac1 and RhoA were known to work antagonistically at the front and the rear of a migrating cell, real-time imaging has shown that their actions are spatially and temporally coordinated at the front of cells during cell protrusion [36–38]. Here, we also measured FRET signals representing real-time activities of Rac1 and RhoA in phagocytes during the uptake of apoptotic cells, hoping that this data may endow more concrete sense of their roles beyond the simple antagonistic functions from overexpression of CA/DN mutants. In addition, these mutants possibly induce robust cytoskeletal phenotypes that then might cover up the real phagocytosis process. We found that cells overexpressing CA RhoA became rigid and phagocytosis is hardly observed in the presence of apoptotic cells in live cell imaging. Cellular processes that occur in a sequential fashion may pass specific irreversible points that are guarded by critical regulatory steps. The molecular events that control efferocytosis are ordered and directional, proceeding from the recognition and engulfment of apoptotic cells to their cellular digestion via lysosomal fusion. Certain crucial points in this phagocytic process, such as closure of the phagocytic cup, must be controlled to ensure that a proper target is engulfed at the correct rate and amount. Based on the unexpected observation that RhoA, a negative regulator, was transiently upregulated at the phagocytic cup immediately prior to ingestion, we hypothesized that this active RhoA, thus shifted Rac1/RhoA balance in the phagocytic cup modulate the critical moment at which a phagocyte decides whether to proceed with eating a meal.

In Fig 2, we observed Rac1/RhoA activity is upregulated by treatment of apoptotic thymocytes but not by fresh thymocytes. Rac1 is activated in multiple sites of membrane with vigorous motility, while RhoA is activated in broad regions with mild and transient way. These cellular phenomena could be caused by PS containing vesicles or soluble factors secreted from the dead cells rather than by direct contact with apoptotic cells. However, the initial Rac1 activation, which can trigger membrane movement via actin polymerization, might play a positive role in the efficient uptake of apoptotic cells. For instance, live cell imaging of fluorescent vaccinia virus showed that extrusion of large membrane blebs are induced by approaching virus particles. The PS coated virus particle as apoptotic mimicry associated with and moved along filopodia to the cell body, where they were internalized [39]. Nagata *et al*. suggested the presence of “the portal of apoptotic cells” based on the observation that apoptotic cells were usually engulfed by the phagocytes’ lamellipodia, where Rac1 was activated. Often, apoptotic cells were engulfed at the same lamellipodial site, where the activated Rac1 was recruited to form phagocytic cups that were comprised of actin patches [14]. The transient RhoA activity observed upon apoptotic cell challenge was an enigma and brought questions; what is the role and is this activity involved in phagocytic process.

To inquire the Rac1/RhoA activity that is directly related with phagocytic process, separately from membrane motility, we monitored Rac1/RhoA FRET activities acquired after contact with an individual apoptotic cell, and chased the activities around the ingested cell (Fig 3). Rac1 activity was observed around the ingested apoptotic cell, promoting the polymerization of actin to enable the membrane to protrude and surround a dead cell, and then decreased once the phagocytic cup was sealed, followed by actin disassembly. Interestingly, the transient activities of RhoA were consistently observed at the phagocytic cup immediately prior to the closure of the phagosomal membrane in multiple cells. RhoA was previously believed to serve as a negative switch for the inhibition of phagocytic processes of apoptotic cells through effector molecule, ROCK [12]. In fact, different effector molecules seem to be involved in RhoA signaling in different phagocytic process depending on targets. For example, the mammalian Diaphanous-related (mDia) formin colocalizes with polymerized actin in the phagocytic cup in αMβ2/CR-3 mediated phagocytosis and interfering with mDia activity inhibits CR3-mediated phagocytosis [16]. On the other hands, inhibitory effects of RhoA is mediated by ROCK and not by mDia formin in Fcγ-receptor mediated phagocytosis and apoptotic cell uptakes in macrophages [12]. Moreover RhoA is activated by direct interaction of surfactant protein (SP) and SIRPα of alveolar macrophages and plays roles to tonically inhibit apoptotic cell engulfment in native lung and this inhibition is fully abrogated by ROCK inhibitor (Y27632) [40]. However, how and when the RhoA/ROCK pathway is activated and how it inhibits the engulfment of apoptotic cells is not fully elucidated [12, 16, 17]. ROCK is a serine/threonine protein kinase and phosphorylates cellular proteins such as myosin light chain II and involved in myosin contractility and cell rigidity to define direction of movement [41] but no target of ROCK in regulation of apoptotic cell engulfment has been identified. ROCK inhibitor-mediated blockade of RhoA signaling dramatically increased the rate and capacity of phagocytosis, rather like fully opening a gate (Fig 4). This might allow uncontrolled phagocytosis of both viable and dying cells as shown that Y27632-treated HMDMs abrogated the inhibitory effects by addition of CD47-Fc. The molecular mechanism of don’t eat me signals, such as CD47, has not been fully elucidated. One potentially important intermediate is the motor protein myosin IIA that is dephosphorylated upon CD47-SIRPα interaction in macrophages, suggesting that self-recognition inhibits contractile engulfment [42]. There is no direct evidence of RhoA/ROCK is involved in “don’t eat me” signaling pathway. Moreover, our results also indicated CD47-Fc does not activate RhoA/ROCK. Thus, further studies are necessary to elucidate molecular basis of the blockade of RhoA function related to don’t eat me signaling. However, our results perhaps suggest a new therapeutic approach for enhancing the ability of immune cells to engulf escaping targets, such as cancer cells.

**Fig 5:**
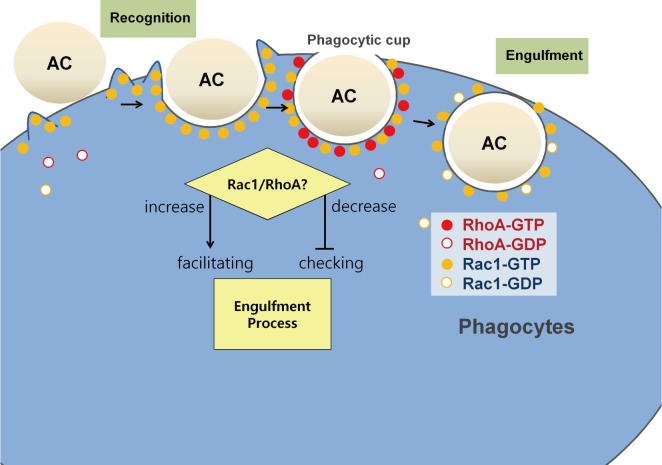
Schematic diagram of Rac1/RhoA balance for engulfment control.

We present novel results suggesting that the Rac1/RhoA balance can modulate the critical point of phagocytosis, the closure of phagocytic cup, via spatiotemporally precise activations and de-activations. When a phagocyte tethers an apoptotic cell via specific interaction of engulfment receptors with PS, Rac1 activity contributes to actin polymerization which is critical for phagocytic cup formation. In contrast, when a phagocyte binds an apoptotic cell, RhoA activity plays a key role in delaying the engulfment process to enforce checking mechanisms and regulate unwanted ingestion (Fig 5). The delay of uptake can be achieved by Rac1 inactivation via ROCK. However, other substrates of ROCK such as myosin light chain 2 [43] and the LIM kinase [44], can be involved in mechanical restraint of uptake by regulation of cytoskeleton organization.

Future work is needed to elucidate how the activities of these Rho-family GTPases are spatiotemporally regulated, and which GEF and GAP regulatory proteins are involved in this modulation. The elaborate control mechanisms of the Rho-family GTPases may form the basis for the ability of various targets and extracellular cues to trigger different modes of phagocytosis.

## Supporting information

S1 Fig Real time activities of Rac1/Rho in L/Stab-2 cells upon challenge with PS and PC liposomes. Representative images of active Rac1 and RhoA biosensor-transfected L/Stab-2 cells treated PS and PC liposomes (Scale bar: 20 μm).
(TIF)

S2 Fig Rho-kinase as an effector for Rho-mediated inhibition of engulfment. (**A**), L/Stab-2 cells were transfected with control vector or RhoA effector specific mutants (G14V, G14V/T37Y, G14V/F39V, and G14V/F39V), and analyzed for engulfment of apoptotic thymocytes. (**B**), The constitutively active RhoA G14V, and constitutively active ROCK mutant (p160ROCK Δ3) were transfected and analyzed for engulfment of apoptotic thymocytes. Rho-kinase inhibitor (Y27632) was treated to the RhoA G14V transfected cells to examine Y27632 overcomes negative regulation of RhoA G14V. The results are means + s.e.m. (n = 3 independent examinations per group. Each examination consists of >500 cells) *P<0.05, **P<0.01, unpaired Student’s *t*-test.
(TIF)

S3 Fig Rho-kinase inhibitor Y27632 promotes phagocytosis of macrophages. **A-F** Untreated (DMSO treated) and Y27632-treated (10 μM) L/Stab-2 cells (**A,B**), bone-marrow derived macrophages (BMDMs) (**C,D**), and peritoneal macrophages (PMs) (**E,F**) were analyzed for engulfment. To monitor engulfment, PS-exposed RBCs were incubated with L/Stab-2 cells and NBD PS-coated beads, with other macrophages. Quantification of phagocytosis was carried out by counting number of engulfed cells or beads per phagocyte under microscopy shown in the representative images (**A,C,E,** right). Bar charts (**A,C,E,** left) show mean phagocytic index + s.e.m. (n = 3 independent examinations per group, Scale bar: 20 μm, Each examination consists of >500 cells). (**B,D,F**) Bar charts show quantification of the percentages of phagocytes that carried the indicated number of PS-coated beads. The relative increase fold of population was shown in the subgraph (the % of DMSO-treated macrophage was set as 1). *P<0.05, **P<0.01, unpaired Student’s *t*-test.
(TIF)

S4 Fig Rac1 or RhoA activities after addition of CD47-Fc. The FRET of Rac1 (A) and RhoA (B) was monitored 5 min after CD47-Fc (1μg/ml) treatment into L/Stab-2 cells (the error bars indicate the s.e.m. of the 3 experiments). The intensities were normalized to the value at 0 min.
(TIF)

S5 Fig Rac1 or RhoA activities after addition of Y27632. The FRET of Rac1 and RhoA was monitored 5 min after Y27632 (30 μM) treatment into L/Stab-2 cells (the error bars indicate the s.e.m. of the 3 experiments). The intensities were normalized to the value at 0 min.
(TIF)

S6 Fig Rac1 or RhoA activities after addition of PS or PC liposomes in Y27632 pretreated phagocytes. The FRET of Rac1 and RhoA was monitored 5 min after PS or PC liposome treatment into L/Stab-2 cells which were pretreated with Y27632 (30 μM for 30 min) (the error bars indicate the s.e.m. of the 3 experiments). The intensities were normalized to the value at 0 min.
(TIF)

S1 Movie FRET ratio imaging of Rac1 activation after addition of apoptotic thymocytes. Rac1 FRET indicator was transfected in L/Stab-2 cells. Vigorous Rac1 activities were monitored in lamellipodia upon membrane movement after treatment of apoptotic thymocytes. Time laps images were recorded in intervals of 30 sec. See also Fig 2A.
(MP4)

S2 Movie FRET ratio imaging of Rac1 activation after addition of fresh thymocytes. (MP4)

S3 Movie FRET ratio imaging of RhoA activation after addition of apoptotic thymocytes. RhoA FRET indicator was transfected in L/Stab-2 cells. Transient RhoA activities were observed with peak of activity around 10 min after treatment of apoptotic thymocytes. Time laps images were recorded in intervals of 30 sec. See also Fig 2A
(MP4)

S4 Movie FRET ratio imaging of RhoA activation after addition of fresh thymocytes. (MP4)

S5 Movie FRET ratio imaging of Rac1 activation in region of interest (ROI) after addition of apoptotic thymocytes. See also Fig 2D.
(MP4)

S6 Movie FRET ratio imaging of RhoA activation in region of interest (ROI) after addition of apoptotic thymocytes. See also Fig 2F.
(MP4)

S7 Movie Activation of Rac1 in the phagocytic cup during engulfment of PS-exposed RBCs. Rac1 FRET indicator was transfected in L/Stab-2 cells. To trigger phagocytosis, L/Stab-2 cells were incubated with damaged RBCs. Rac1 activity was found around ingested damaged RBCs and decreased once the phagocytic cup was sealed. Time laps images were recorded in intervals of 30 sec. See also Fig 3B and 3C.
(MP4)

S8 Movie Activation of RhoA in the phagocytic cup during engulfment of damaged RBCs. RhoA FRET indicator was transfected in L/Stab-2 cells. To trigger phagocytosis, L/Stab-2 cells were incubated with PS-exposed RBCs. The RhoA was transiently activated at the phagocytic cup before the phagosomal membrane closure. Time laps images were recorded in intervals of 30 sec. See also Fig 3D and 3E.
(MP4)

S9 Movie The engulfment of PS-exposed RBCs by L/Stab-2 cells under DMSO treatment. DMSO was pre-treated 1 h before phagocytosis. L/Stab-2 cells engulfed about 5 PS-exposed RBCs for 3 h. Time laps images were recorded in intervals of 1 min. See also Fig 4A.
(MP4)

S10 Movie The engulfment of PS-exposed RBCs by L/Stab-2 cells under ROCK inhibitor, Y27632 treatment. Y27632 was pre-treated 1 h before phagocytosis. Vigorous engulfment was observed for 3 h when Rho kinase was blocked as described in Fig 4A. Time laps images were recorded in intervals of 1 min. See also Fig 4A.
(MP4)
