# Supplementary figures and images for: Coordinated balance of Rac1 and RhoA plays key roles in determining phagocytic appetite
Source: PLoS One. 2017 Apr 4;12(4):e0174603. doi: 10.1371/journal.pone.0174603 (PMC5380344; doi:10.1371/journal.pone.0174603)

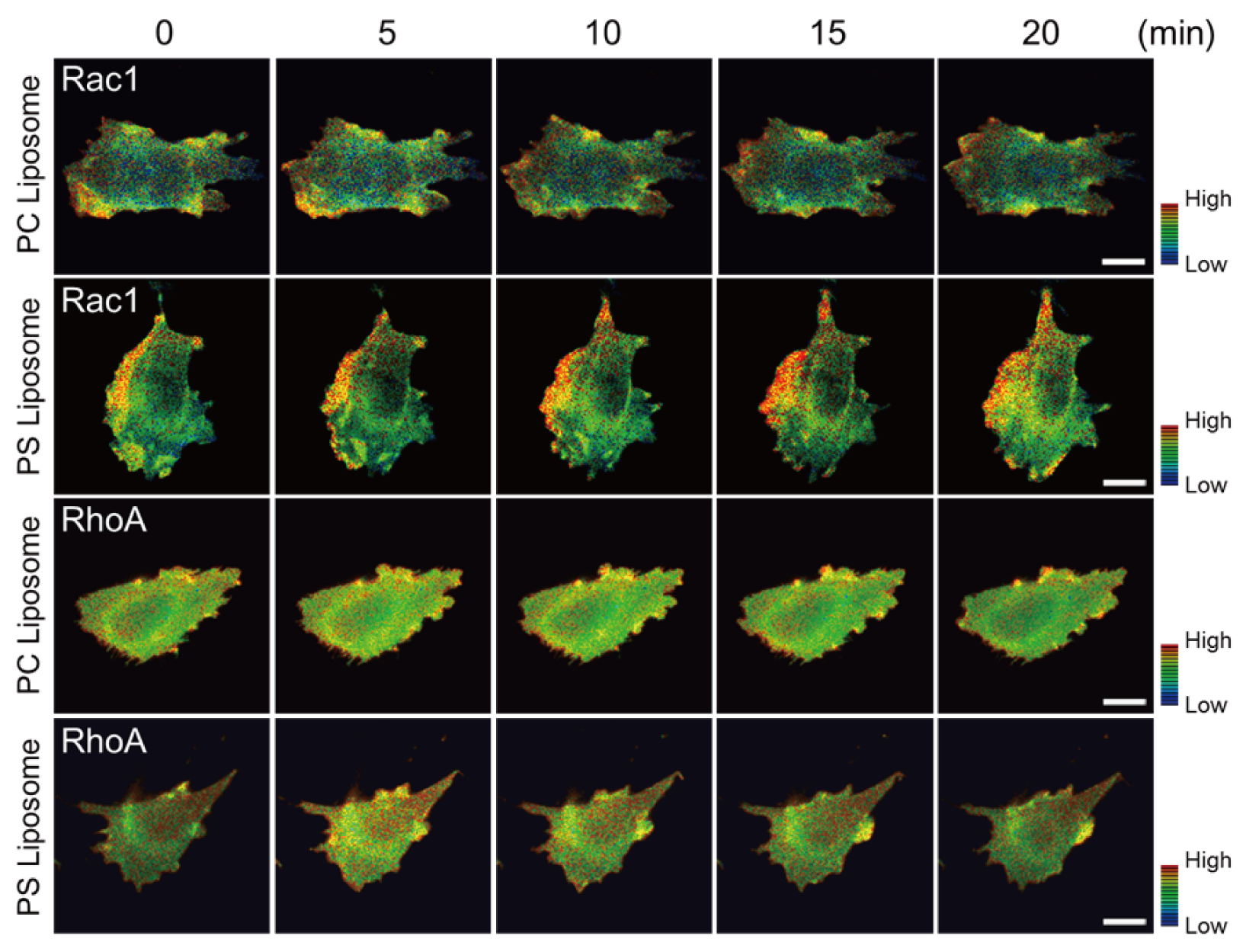

Supplement: S1 Fig — Representative images of active Rac1 and RhoA biosensor-transfected L/Stab-2 cells treated PS and PC liposomes (Scale bar: 20 μm). (TIF) [file pone.0174603.s001.tif]

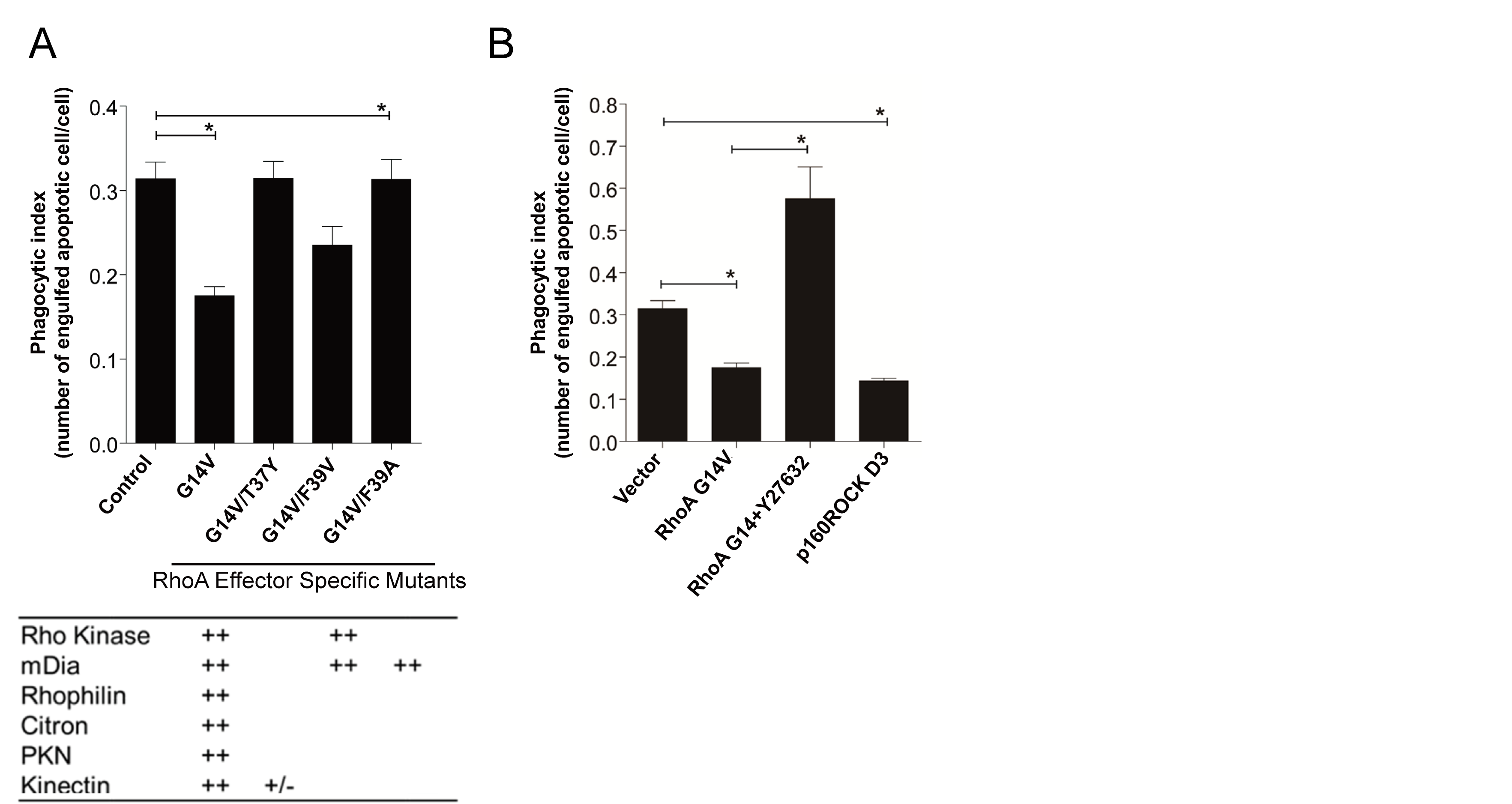

Supplement: S2 Fig — (A), L/Stab-2 cells were transfected with control vector or RhoA effector specific mutants (G14V, G14V/T37Y, G14V/F39V, and G14V/F39V), and analyzed for engulfment of apoptotic thymocytes. (B), The constitutively active RhoA G14V, and constitutively active ROCK mutant (p160ROCK Δ3) were transfected and analyzed for engulfment of apoptotic thymocytes. Rho-kinase inhibitor (Y27632) was treated to the RhoA G14V transfected cells to examine Y27632 overcomes negative regulation of RhoA G14V. The results are means + s.e.m. (n = 3 independent examinations per group. Each examination consists of >500 cells) *P<0.05, **P<0.01, unpaired Student’s t-test. (TIF) [file pone.0174603.s002.tif]

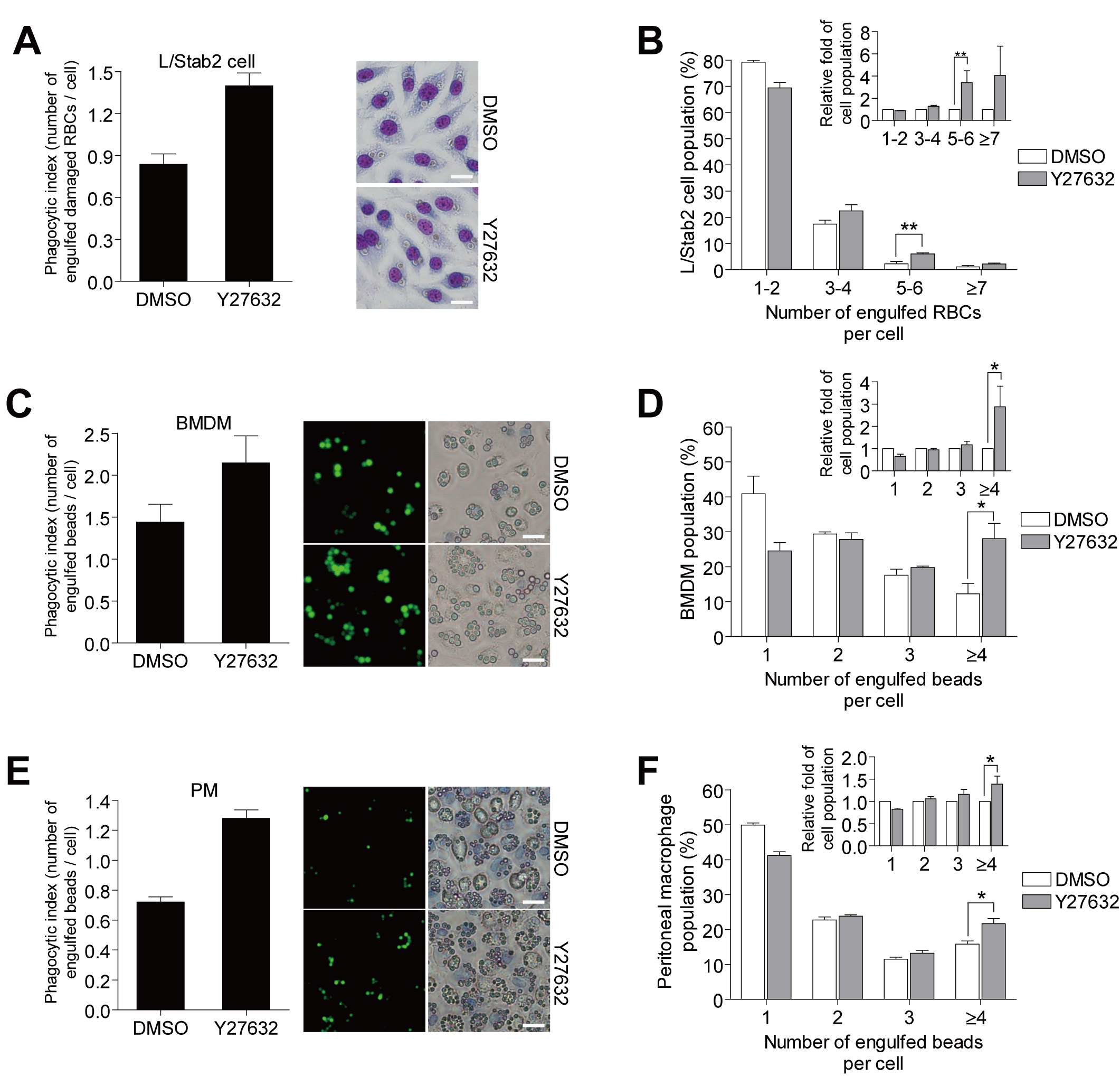

Supplement: S3 Fig — A-F Untreated (DMSO treated) and Y27632-treated (10 μM) L/Stab-2 cells (A,B), bone-marrow derived macrophages (BMDMs) (C,D), and peritoneal macrophages (PMs) (E,F) were analyzed for engulfment. To monitor engulfment, PS-exposed RBCs were incubated with L/Stab-2 cells and NBD PS-coated beads, with other macrophages. Quantification of phagocytosis was carried out by counting number of engulfed cells or beads per phagocyte under microscopy shown in the representative images (A,C,E, right). Bar charts (A,C,E, left) show mean phagocytic index + s.e.m. (n = 3 independent examinations per group, Scale bar: 20 μm, Each examination consists of >500 cells). (B,D,F) Bar charts show quantification of the percentages of phagocytes that carried the indicated number of PS-coated beads. The relative increase fold of population was shown in the subgraph (the % of DMSO-treated macrophage was set as 1). *P<0.05, **P<0.01, unpaired Student’s t-test. (TIF) [file pone.0174603.s003.tif]

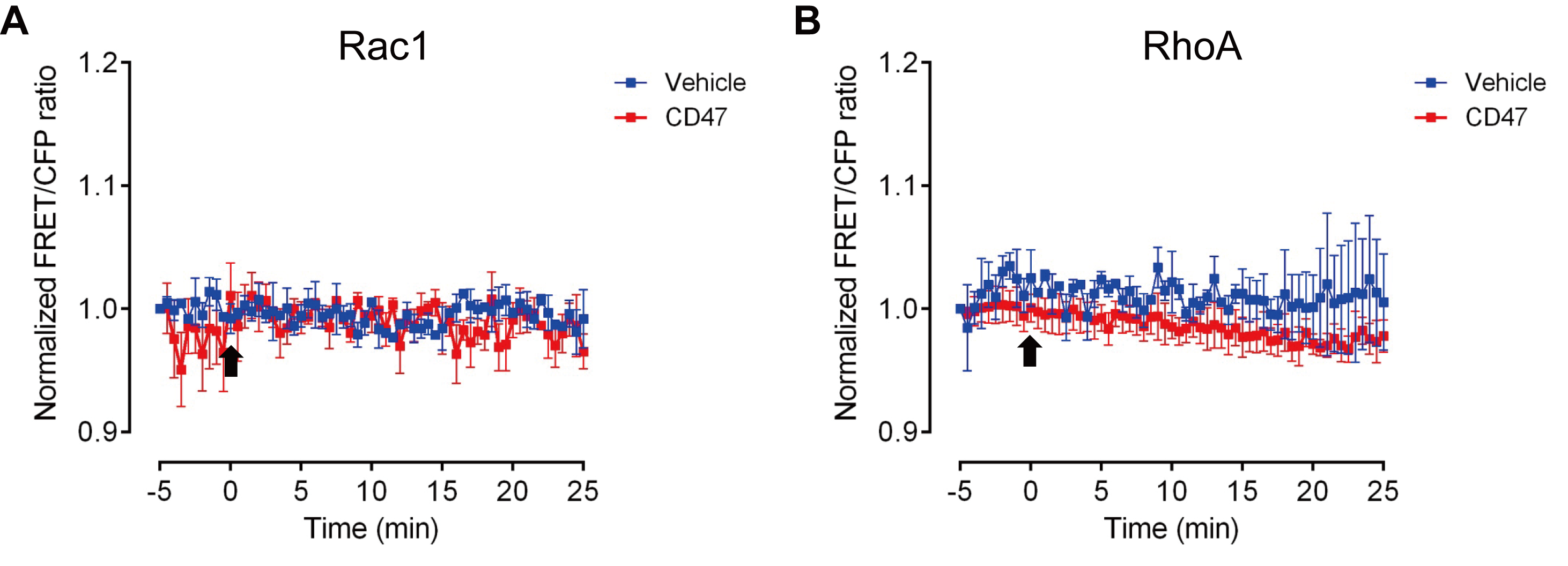

Supplement: S4 Fig — The FRET of Rac1 (A) and RhoA (B) was monitored 5 min after CD47-Fc (1μg/ml) treatment into L/Stab-2 cells (the error bars indicate the s.e.m. of the 3 experiments). The intensities were normalized to the value at 0 min. (TIF) [file pone.0174603.s004.tif]

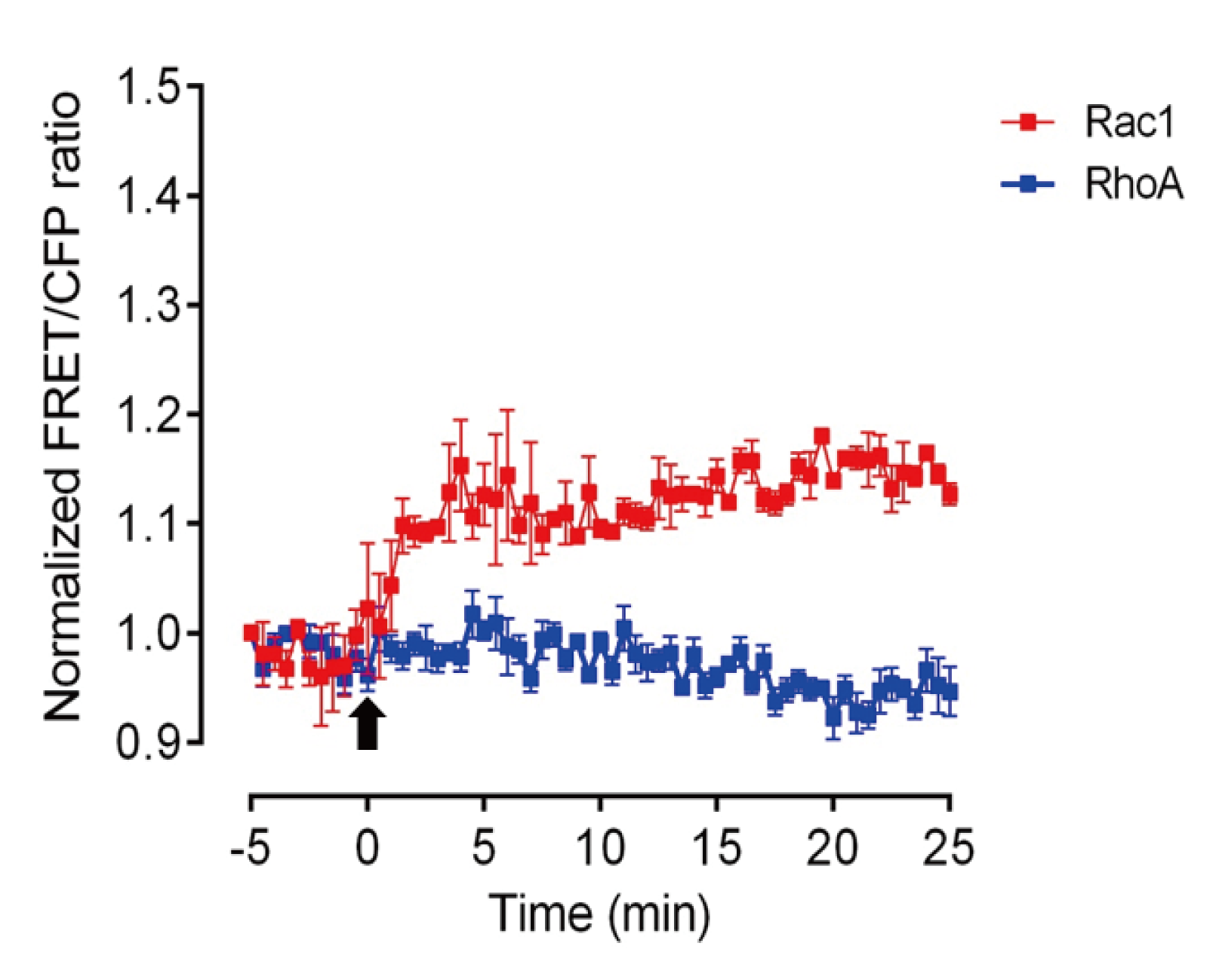

Supplement: S5 Fig — The FRET of Rac1 and RhoA was monitored 5 min after Y27632 (30 μM) treatment into L/Stab-2 cells (the error bars indicate the s.e.m. of the 3 experiments). The intensities were normalized to the value at 0 min. (TIF) [file pone.0174603.s005.tif]

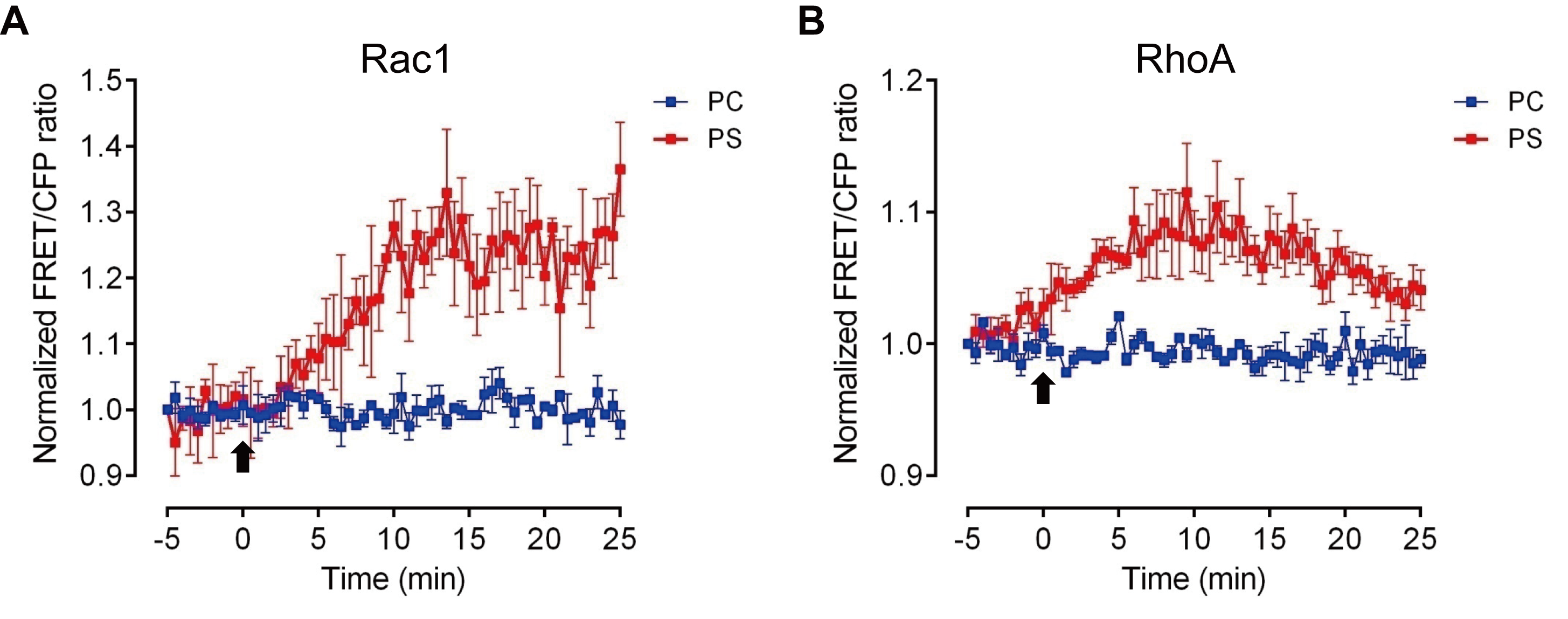

Supplement: S6 Fig — The FRET of Rac1 and RhoA was monitored 5 min after PS or PC liposome treatment into L/Stab-2 cells which were pretreated with Y27632 (30 μM for 30 min) (the error bars indicate the s.e.m. of the 3 experiments). The intensities were normalized to the value at 0 min. (TIF) [file pone.0174603.s006.tif]
